# Beneficial effects of IL-4 and IL-6 on rat neonatal target cardiac cells

**DOI:** 10.1038/s41598-020-69413-0

**Published:** 2020-07-23

**Authors:** Camila Zogbi, Nathalia C. Oliveira, Débora Levy, Sergio P. Bydlowski, Vinicius Bassaneze, Elida A. Neri, Jose E. Krieger

**Affiliations:** Lab Genetics & Mol Cardiology/LIM 13, Heart Institute (InCor), University of São Paulo Medical School, Av Dr Eneas C Aguiar 44, Sao Paulo, SP 05403-000 Brazil

**Keywords:** Cell growth, Cardiac regeneration, Data processing

## Abstract

The nature of the early post-natal immune response in rodents appears to influence cardiac regeneration even though the underlying molecules remain poorly understood. Consistent with this idea, we show now significant changes in the expression of immune and cell movement gene pathways in heart samples from 1- and 7-day-old rats with ventricle resection. We then tested whether conditioned media from adult M2 anti-inflammatory macrophages target neonatal cardiac cells to a pro-regenerative like phenotype compared to the M1 pro-inflammatory macrophages. We found that M2 compared to M1 macrophage-conditioned media upregulates neonatal cardiomyocyte proliferation, suppresses myofibroblast-induced differentiation and stimulates endothelial cell tube formation. Using a cytokine array, we selected four candidate cytokine molecules uniquely expressed in M2 macrophage-conditioned media and showed that two of them (IL-4 and IL-6) induce endothelial cell proliferation whilst IL-4 promotes proliferation in neonatal cardiomyocytes and prevents myofibroblast-induced collagen type I secretion. Altogether, we provided evidence that adult M2 macrophage-conditioned media displays a paracrine beneficial pro-regenerative response in target cardiac cells and that IL-4 and IL-6 recapitulate, at least in part, these pleiotropic effects. Further characterization of macrophage immune phenotypes and their secreted molecules may give rise to novel therapeutic approaches for post-natal cardiac repair.

## Introduction

Adult cardiac repair occurs by mending damage to scar tissue without significant cardiomyocyte regeneration, leading to increased long-term morbidity and mortality. The healing process schematically involves an inflammatory, proliferative and maturation phase as observed in vascular models^1,2^. Local inflammation recruits circulating cells to clear cell debris in preparation for the proliferative phase. The second wave of inflammation is responsible for secreting growth factors and recruiting/activating reparative cells to preserve the structural integrity of the chamber and prevent wall rupture^3–6^. Leukocyte subsets such as pro- (M1) and anti-inflammatory (M2) macrophages play an important role in repairing the injured heart, and there may be differences in their profile between neonates and adults^7–10^. One may reason that the adult mammalian immune system restrains regenerative capacity, while the immature inflammatory response supports regeneration in lower animals after injury^9,10^. Alternatively, the regenerative response is context-dependent and influenced by a number of factors including the ontogenetic development of the target cardiac cells (e.g. cardiomyocytes, fibroblasts, endothelial and resident and non-resident immune cells, to name a few) and the balance of the macrophage subtype activation with unique phenotypes, such as, but not limited to, M1 and M2 macrophages. Indeed, adaptive and innate immune systems undergo severe alterations and maturation during the neonatal period, which coincides with the developmental loss of myocardium regenerative capacity^11–13^. In neonates, macrophage influx after injury is essential for repair^13^, whereas predominant scar tissue formation in adults may be related to the source and phenotype of inflammatory macrophages^7^.

In the present study, we used first global gene expression analysis in the in vivo new-born rat apex resection model to assess the immune-inflammatory gene pathways in the pro-regenerative response of 1-day-old ventricle-resected rats compared to the predominant repair response observed in 7-day-old ventricle-resected animals, which is similar to that observed in adults^14,15^. Indeed, we observed that the inflammatory pattern of activation is different between 1-day-old and the 7-day-old resected rats and demonstrated that the adult M2 macrophage-conditioned media recapitulates the pro-regeneration effects on target cardiac cells suggesting that the quality of the inflammation may play a role. Moreover, we showed that the M2 candidate secreted cytokines IL-4 and IL-6 recapitulate, at least in part, these pleiotropic regenerative effects, suggesting that key inflammatory mediators display a regenerative-like response in cardiac target cells.

## Results

### Age-dependent induced changes are the overwhelming factor in the first two post-natal weeks

**Figure 1:**
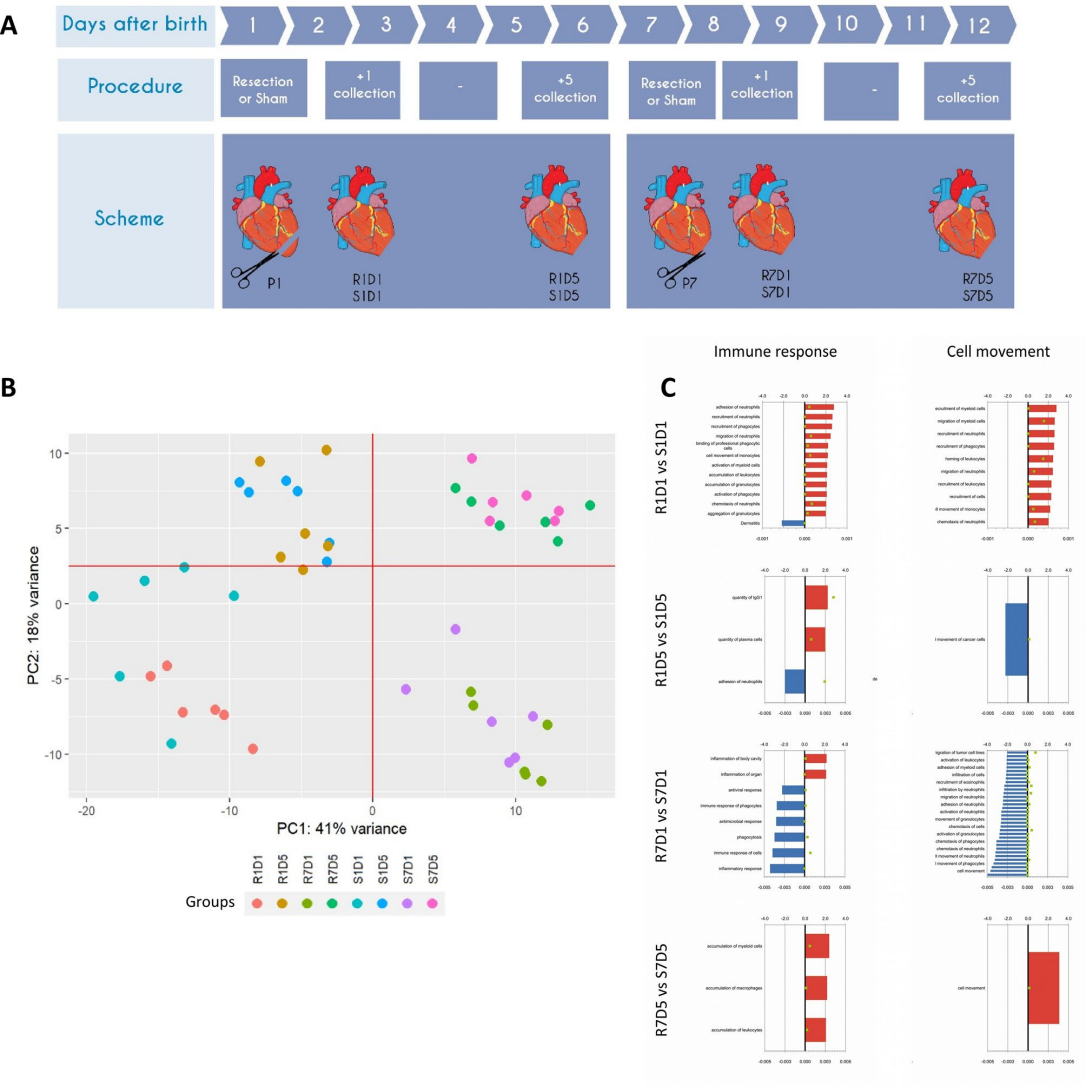
Total RNA sequencing of resected neonatal hearts. (**A**) Experimental design scheme for total RNA sequencing. Cardiac apex resection or false surgery was performed in 1-day-old and 7-day-old rats, and samples were collected one (D1) or five (D5) days after the procedure. We created this figure in the Mind the Graph platform (www.mindthegraph.com). (**B**) Only the apex portion (1/3 of the heart) was considered for the experiment; in the PCA diagram, samples were clustered considering three main parameters: age at time of procedure, procedure and time post-procedure. The more prevalent responses were age at time of procedure (x axis, corresponds to 42% of the variance) followed by time post-procedure (y axis, corresponds to 19% of the variance). (**C**) Biological processes and pathways related to the inflammatory response and cellular movement in comparison to their respective sham-operated groups at different time points (R1D1/S1D1; R1D5/S1D5; R7D1/S7D1 and R7D5/S7D5) through ingenuity enrichment analysis. Red and blue bars show activation and inactivation of pathways, respectively, showing that the overall inflammatory responses are contrasting between neonatal development times.

The cardiac neonatal period (from day one to day twelve, Fig. 1A) is characterized by intense gene expression changes reflecting cell proliferation, differentiation and biological processes that support the activities in the evolving heart during ontogeny. Intriguingly, even after an important stimulus, that is, left ventricle resection in which approximately 16% of the total heart is removed, as assessed by pre- and post-MRI, the global molecular signatures appear to be mainly correlated with the ontogeny of the organ and not with the experimental procedure, as shown by an unsupervised analysis using the primary component analysis (PCA) of the RNASeq data (Fig. 1B).

We then performed enrichment analyses and identified molecular signatures associated with the immune response and cell migration that differed between the sham and experimental groups for each studied period (Fig. 1C). These data show activations of inflammation and cell migration pathways in one-day-old resected rats compared to the respective sham-operated animals (R1D1 vs. S1D1; Fig. 1C). In addition, the activated responses were highly contrasting between 1- (P1) and 7-day-old rats (P7; Fig. 1C) in response to the same stimulus, namely, ventricle resection. Collectively, these results highlight that the inflammation in response to the lesion is different, even though the groups are just 6 days apart, and it is tempting to speculate that this unique pattern of response may influence the capacity to regenerate cardiomyocytes.

### Macrophages are crucial for heart regeneration in P1

**Figure 2:**
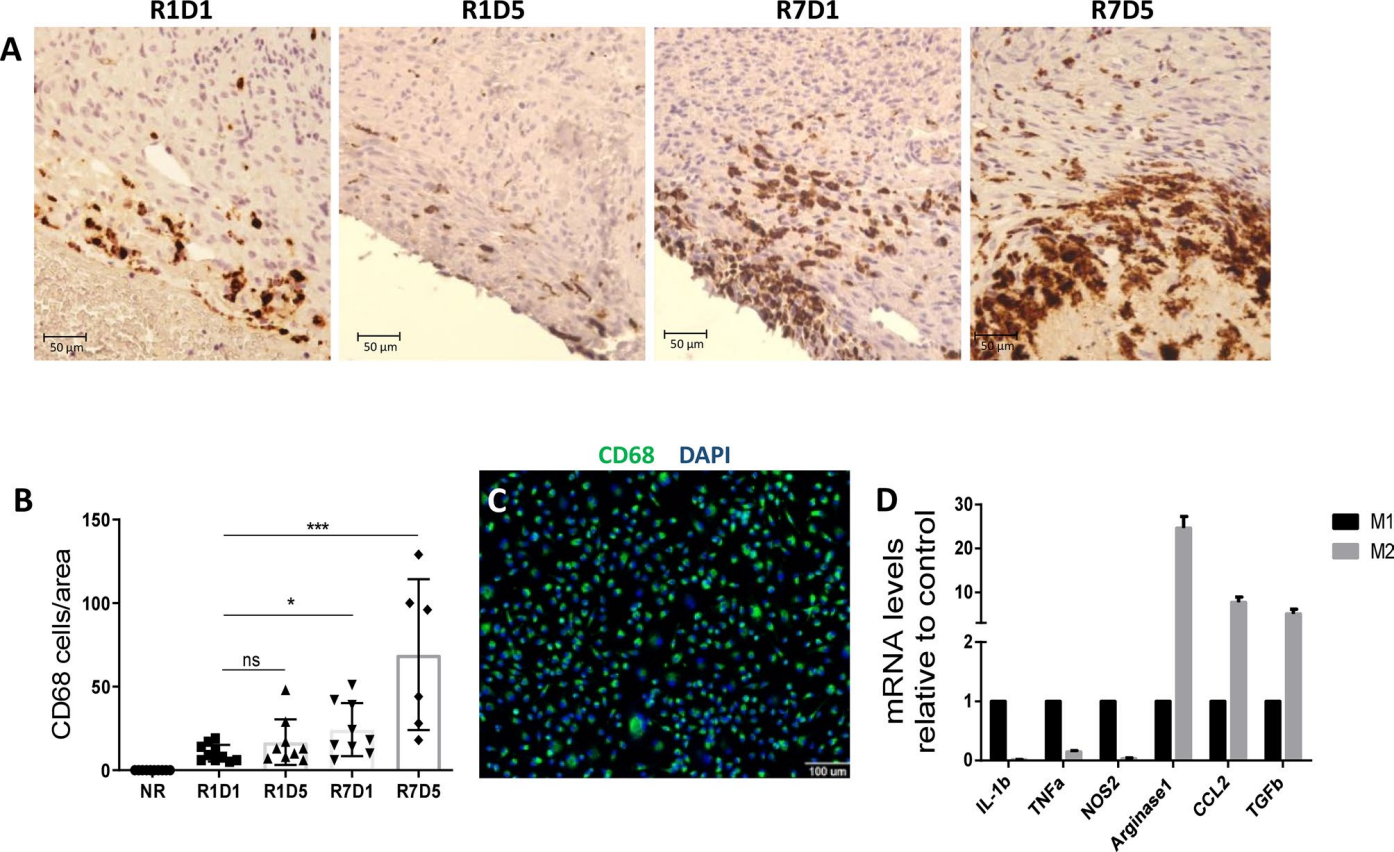
Macrophage infiltration after apex resection. (**A**) Macrophage infiltration evaluated (mean ± SD) by CD68-positive cell staining in the apex areas of R1D1 (n = 7), R1D5 (n = 7), R7D1 (n = 7) and R7D5 (n = 6). (**B**) Bars show total CD68-positive cells normalized by heart tissue area, considering that non-resected rats do not display macrophage infiltration in the apex area and that CD68 infiltration increases with time. Values are relative to P1D1. ns = non-significant; *p-value ≤ 0.05 and ***p-value ≤ 0.001. (**C**) Adult bone marrow-derived macrophages cultivated for 7 days were stained for CD68 and then polarized towards M1 and M2 profiles. (**D**) The gene expression pattern of polarized macrophages was analysed by real-time PCR. M1 polarized macrophages were confirmed for IL-1b, TNF-a and NOS2 gene expression (black bars), and M2 polarized macrophages were confirmed for Arginase 1, CCL2 and TGF-b (grey bars). Scale bars = 50 µm e 100 µm.

Upon tissue insult, macrophages are the main innate immune cells that immediately invade damaged tissue, consistent with this idea. We showed that CD68-positive macrophages invaded the resected hearts shortly after injury (Fig. 2A). Macrophage invasion appeared to increase as the animals aged from day 2 through day 12 (Fig. 2B). Considering that differences in the outcome might rely on the macrophage source and phenotype, we hypothesized that the pro-reparative subpopulation, that is, the anti-inflammatory M2 macrophages, is associated with the repair/regeneration responses seen in P1 compared with repair mainly through scar formation observed in P7 rats.

We failed to isolate and characterize the neonatal heart infiltrated macrophages due to the limited availability of reagents to be tested on rats, unlike in mice, as discussed in the methods. Alternatively, we cultured and polarized rat adult bone marrow-derived macrophages (BMDMΦs) into both pro- and anti-inflammatory phenotypes for proof of concept studies (Fig. 2C). As shown in Fig. 2D, we confirmed the polarization of M1 and M2 phenotypes by assessing the expression of IL-1β, TNF-α and NOS2, as well as Arginase 1, CCL2 and TGF-β by qRT-PCR, respectively.

### M2-conditioned media recapitulates pro-regenerative effects on cardiac cells under stress

**Figure 3:**
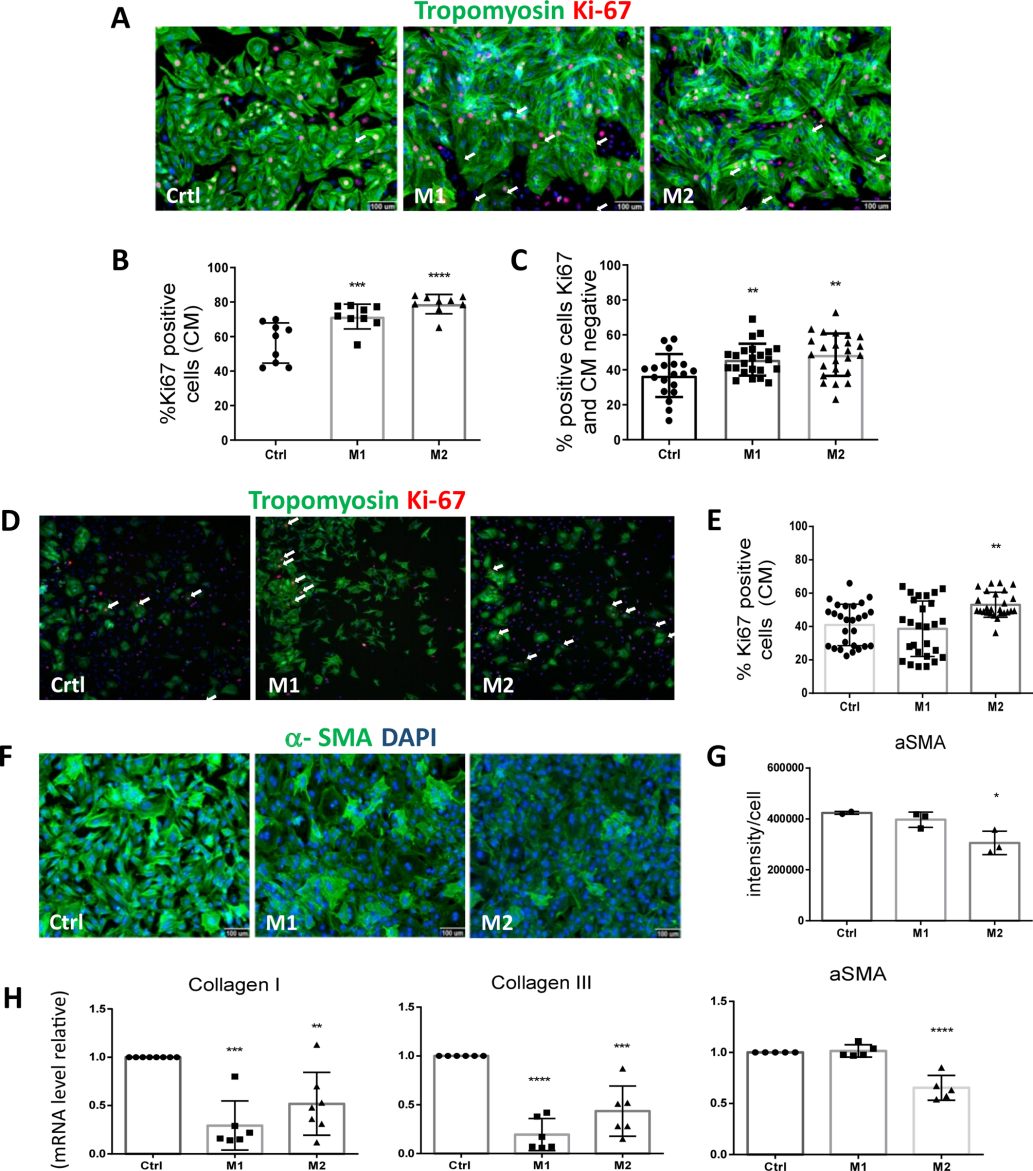
M2-conditioned media increase cardiomyocyte proliferation and reduce α-SMA gene and protein expression in TGF-β-stimulated fibroblasts. (**A**) Representative images of HCS of cardiomyocytes under normoxia treated with M1 and M2 compared to control culture medium. Staining for tropomyosin (green), Ki67 (red) and nuclei with DAPI (blue). Phenotypic parameters were retrieved for each single-cell identified and only cardiomyocytes were included in the analysis. Scale bars = 100 µm. (**B**) Percentage of Ki67-positive cardiomyocytes by HCS (A) (N = 8–9). *** p-value ≤ 0.001 ****p-value ≤ 0.0001. (**C**) Percentage of Ki67-positive cardiomyocyte-negative cells by HCS. Only cardiomyocytes-negative cells were included in the analysis (A). (**D**) Representative images of HCS of cardiomyocytes under hypoxia treated with M1 and M2 compared to control culture medium. Scale bars = 100 µm. (**E**) Percentage of Ki67-positive cardiomyocytes by HCS (D) (N = 29). **p-value ≤ 0.01. (**F**) Representative images of HCS of TGF-β1-stimulated fibroblasts treated with M1- and M2-conditioned media after 24 h of treatment. Staining for α-SMA (green) and DAPI (blue). Scale bars = 100 µm. (**G**) Mean expression of α- SMA per TGF-β1-stimulated fibroblasts given by the average of α- SMA fluorescence intensity (B) (N = 3). (**H**) Relative expression determined by Real-time PCR analysis of TGF-β-stimulated fibroblasts treated with M1- and M2-conditioned media after 24 h of treatment. GAPDH was used as housekeeping gene and the results are displayed as 2^-ΔΔCt^ , mean ± SEM. **p-value ≤ 0.01, ***p-value ≤ 0.001 and ****p-value ≤ 0.0001. (N = 5–8).

The effects of conditioned media soluble factors from both M1 and M2 macrophages on target cardiac cells were evaluated. We treated 1-day-old cardiomyocytes with both conditioned media for 24 h under stress culture conditions. Both M1 and M2 sustained neonatal cardiomyocyte proliferation under basal culture conditions (Fig. 3A,B). However, under hypoxic culture conditions, only M2-conditioned media increased cardiomyocyte proliferation (Fig. 3D,E). In addition to cardiomyocytes, endothelial cells and cardiac fibroblasts are also targeted by macrophages^16^. However, fibroblasts are pivotal in the remodelling of the architecture and support of the myocardium and are associated with the onset and progression of fibrotic processes. Neonatal fibroblast cells were cultured in the presence of 10 ng/ml TGF-β alone or in combination with conditioned media for 48 h. Likewise, both M1- and M2-conditioned media supported cardiac fibroblast proliferation under basal culture conditions (Fig. 3C) and suppressed the expression of both collagen types I and III in TGF-β-stimulated myofibroblasts (Fig. 3H). Nevertheless, only M2-conditioned media decreased TGF-β-induced myofibroblast differentiation/maturation in vitro, as evaluated by the low intensity of α-SMA protein expression (Fig. 3F,G) and the reduction in α-SMA gene expression (Fig. 3H). Altogether, these paracrine effects of M2 macrophage soluble factors showed beneficial pro-reparative/regeneration responses and warranted a search for possible candidates.

### The candidates IL-4 and IL-6 recapitulate key beneficial effects of M2-conditioned media in target cardiac cells

**Figure 4:**
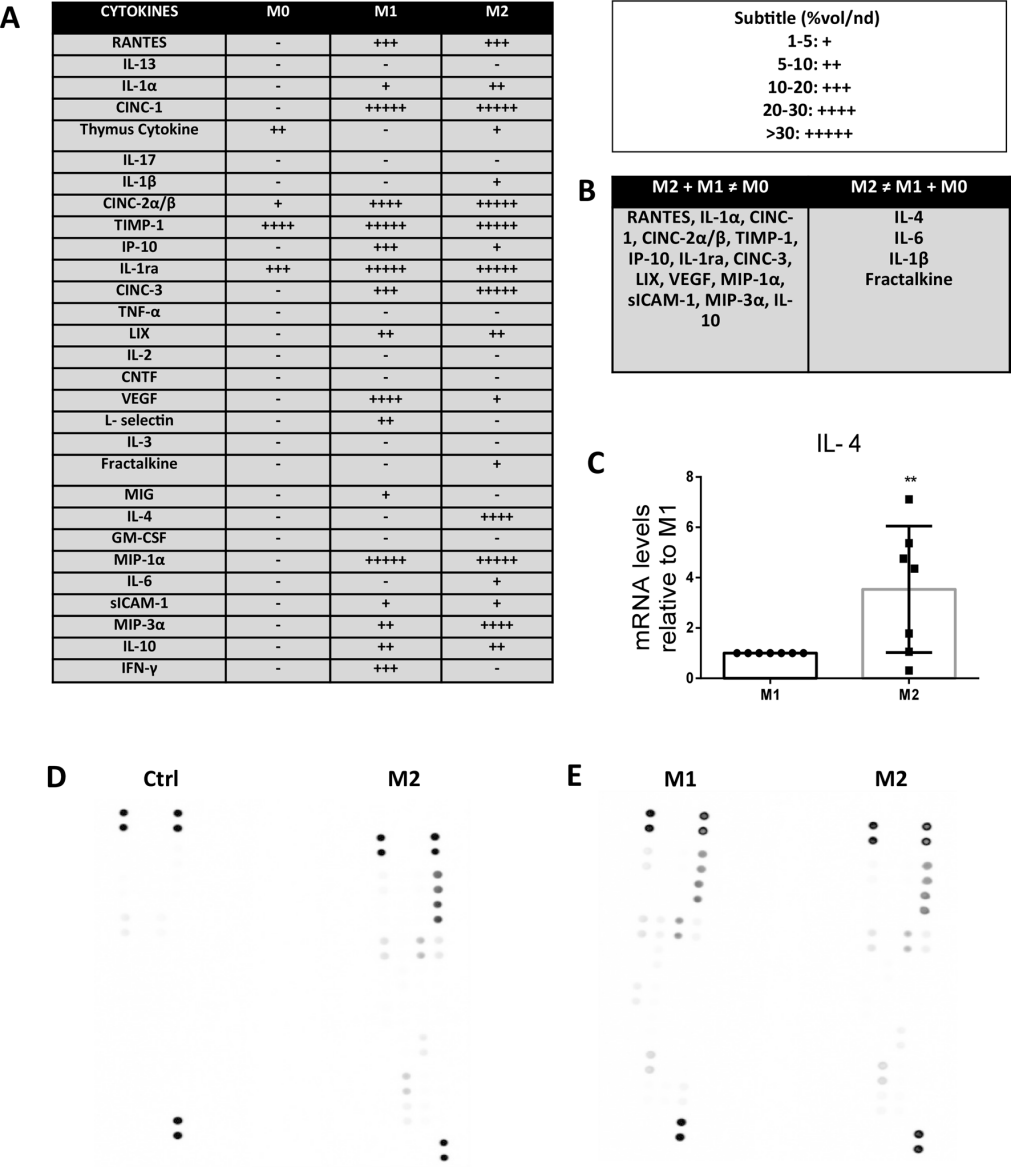
Protein and gene expression of M2 macrophages. (**A**) Cytokine array panel randomization of M0-, M1- and M2-conditioned media expressed cytokines. (**B**) Cytokines expressed from both M2 and M1, different from M0 and exclusively expressed M2 candidate cytokines, such as IL-4, IL-6, IL-1β and fractalkine. (**C**) Real-time PCR data analysis of the IL-4 gene confirmed that M2 macrophages express IL-4 after 48 h of polarization. **p-value ≤ 0.01. The membranes were generated and compared as pairs: (**D**) macrophage control means vs M2 and (**E**) M1 vs M2. The M1- and M2-conditioned media were obtained from the combination of independent polarization experiments (n = 7).

**Figure 5:**
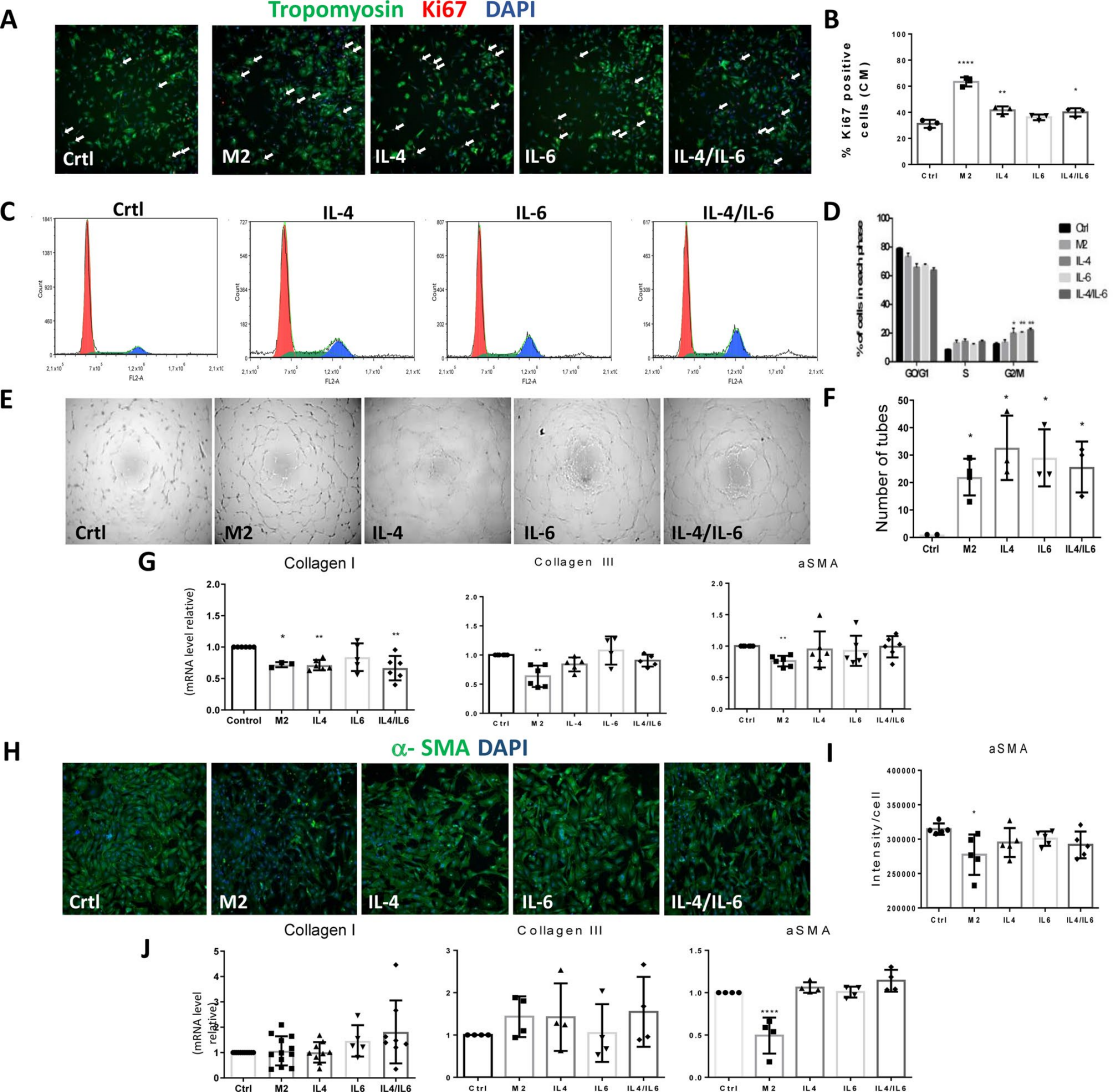
Candidate cytokines IL-4 and IL-6 recapitulate part of the pro-repair/regeneration responses. (**A**) Representative images of HCS of cardiomyocytes treated with M2, IL-4, IL-6 and IL-4/IL-6 compared to control culture medium. Scale bars = 100 µm. (**B**) Percentage of Ki67-positive cardiomyocytes by HCS (A). **p-value ≤ 0.01 and ****p-value ≤ 0.0001. (N = 3). (**C**) DNA content was measured by flow cytometry by tracing the fluorescence intensity of HUVECs treated with M2, IL-4, IL-6 and IL-4/IL-6. (**D**) The graphical representation of (**C**). **p-value ≤ 0.01. (**E**) Images show the tube formation of HUVECs treated with Crtl culture medium, M2, IL-4, IL-6 and IL-4/IL-6. (**F**) Quantification of the tubes formed of HUVECs treated (**E**). **p-value ≤ 0.01. (**G**) Real-time PCR data analysis of a-SMA, collagen I and collagen III in TGF-β1-stimulated fibroblasts treated with M2, IL-4, IL-6 and IL-4/IL-6 compared to control culture medium. (N = 3–6). **p-value ≤ 0.01. (**H**) Representative images of high-content screening of TGF-β1-stimulated fibroblasts treated with M2, IL-4, IL-6 and IL-4/IL-6 compared to control culture medium. Scale bars = 100 µm. (**I**) Quantification of α-SMA fluorescence intensity in the TGF-β1-stimulated fibroblasts (H). (**J**) Real-time PCR data analysis of a-SMA, collagen I and collagen III on myofibroblasts from the infarcted area. ****p-value ≤ 0.0001. N = 3–12.

We performed a cytokine array to assess M2-conditioned media candidate soluble factors. Using a comparative analysis among M0 (9-day culture of BMDMφs, without polarization), M1 and M2 expressed cytokines, we selected only cytokines exclusively expressed in M2-conditioned media (Fig. 4), such as IL-4, IL-6, IL-1β and fractalkine (Fig. 4A,B). Since IL-4 is the main M2-expressed cytokine, and it was the protein used to polarize macrophages towards an anti-inflammatory phenotype, we showed that even after 48 h of polarization and without IL-4 addition to the culture medium, M2 macrophages expressed IL-4 (Fig. 4C). As a proof of concept, we considered IL-4 and IL-6 as candidates to evaluate whether candidate cytokines recapitulate the effects of M2-conditioned media. Cardiomyocytes were treated with 20 ng/ml IL-4 and 10 ng/ml IL-6 for 24 h. Similar to M2-conditioned media, IL-4 increased neonatal cardiomyocyte proliferation under stress culture conditions, while IL-6 alone failed to stimulate it (Fig. 5A,B). On the other hand, both candidate cytokines IL-4 and IL-6 increased endothelial cell proliferation when evaluated by changes in G2/M cell cycle progression after 24 h of culture (Fig. 5C,D), while both cytokines and M2-conditioned media increased tube formation in HUVECs when evaluated by the number of tubes formed after 6 h of culture (Fig. 5E,F). Additionally, the IL-4 cytokine reduced collagen type I gene expression in TGF-β-stimulated myofibroblasts in vitro (Fig. 5G) but failed to decrease both collagen type III expression and α-SMA gene and protein expression (Fig. 5G,I). Therefore, we observed the protective effect of M2-conditioned media on TGF-β-stimulated myofibroblasts in vitro (Fig. 5G). We evaluated the capacity of reducing α-SMA expression in cardiac myofibroblasts when isolated from 15-day infarcted adult rats. Remarkably, only M2-conditioned media decreased α-SMA gene expression in myofibroblasts from infarcted adult rats, while collagen type I and III expression remained unchanged (Fig. 5J).

Collectively, our results show the strong pleiotropic effects of M2-conditioned media soluble factors on the pro-regeneration outcomes of target cells and that IL-4 and IL-6 individually recapitulate key beneficial effects on cardiac target cells with the potential to influence cardiac repair. Furthermore, the anti-inflammatory stimulation profile is beneficial for inducing positive responses and may explain the scar-free regeneration of P1 resected neonates.

## Discussion

Here, we provide evidence that anti-inflammatory macrophage soluble factors display pro-regenerative effects on target neonatal cardiac cells. We speculate that the pattern of inflammatory response contributes to the regenerative potential in neonatal cardiac tissue and provide evidence that adult macrophage soluble factors recapitulate, at least in part, the capacity to elicit the pro-regenerative phenotypes in neonatal target cardiac cells. A great body of evidence suggests that the mammalian immune response is quantitatively and qualitatively different in neonates and that maturation of this system correlates with a decline in cardiac regenerative potential^17,18^. To test this hypothesis, we analysed RNASeq data (Fig. 1A,B) using a non-supervised cluster analysis to show that age-dependent expression changes associated with ontogeny of the organ are the predominant driver in this period. Previous data suggest that neonates generate less inflammation after tissue damage associated with deficiencies in immune cell infiltration and the regulatory cytokine milieu under the robust influence of IL-10^19^. Our data, however, indicate that there is robust inflammatory gene pathway activation in 1-day-old resected rats compared to paired sham-operated rats (Fig. 1C) and that macrophages do indeed infiltrate the heart tissue shortly after injury in P1 animals (Fig. 2A). Aurora and colleagues showed that the predominant profile of macrophages in 1-day-old mice is anti-inflammatory M2-like and that they are crucial for heart regeneration^13^. More recently, Simoes et al. in an elegant series of studies provided evidence that adult macrophages can directly contribute to fibrosis during cardiac repair, even though they have not characterized the immune phenotype of the macrophages, which might have allowed to assess whether the balance of the macrophage activation response also plays a role in the neonatal and adult settings as our data suggest^20^. Additionally, IL-13, an anti-inflammatory cytokine, has been shown to be required to support cardiac regeneration in 1-day-old mice following apex resection. Using IL-13 knockout animals, Wodsedalek and colleagues showed that regeneration was impaired^21^.

The main difficulty in testing the hypothesis that P1 infiltrated macrophages displayed anti-inflammatory and pro-regeneration properties was the isolation of the infiltrated macrophages since there are no specific rat membrane antibodies as observed for mice and high neonatal cardiomyocyte autofluorescence. Alternatively, we developed a specific differentiation/polarization protocol of bone-marrow-derived macrophages for proof of concept assessment and showed for the first time the direct effect of adult M2-conditioned media on modifying the phenotype of target cardiac cells towards a regenerative response with increasing cardiomyocyte proliferation under normoxia (Fig. 3A,B) and hypoxia, mimicking the infarcted tissue (Fig. 3D,E); decreasing collagen types I and III expression (Fig. 3H); and decreasing α-SMA gene and protein expression on both TGF-β-induced myofibroblasts and isolated cardiac myofibroblasts from rats that have undergone myocardial infarction (Fig. 3F–H; Fig. 5G–J). These results highlight the fact that adult M2-macrophage secreted soluble factors that can act upon permissive neonatal cardiac cells to elicit pro-regenerative phenotypes. In addition, M2-conditioned media favoured tube formation of HUVECs (Fig. 5E,F).

Previous studies focused on the contribution of macrophages to wound repair with their role as scavenger cells to remove cellular debris and other apoptotic cells following tissue injury^7,22^. Now, a more complex scenario has emerged in which monocytes and macrophages may also play a role in modulating fibrosis and tissue regeneration^7,23^ . The literature suggests that M2 macrophages secrete a variety of soluble factors including neurotransmitters, hormones and growth factors^24^, and molecules such as insulin-like growth factor-1 (IGF-1), which can influence tissue regeneration^25^ and have a role in sustaining M2 activation^26^. These observations are consistent with the lack of tissue regeneration seen in mice that were experimentally depleted in macrophage favouring a predominant repair response via scar formation^13^. Aurora et all observed different subtypes of macrophages in neonatal mice following myocardial infarction, but it remains to be determined whether the neonatal macrophages are unique compared to the adult ones and/or if the pattern of macrophage activation differs. Our data, showing that adult M2 macrophage soluble factors suffice to elicit a pro-regenerative program in neonatal cardiac cells, suggest that independent of their similarities, neonatal and adult macrophages share these pro-regenerative properties. It is important to emphasize that the neonatal target cardiac cells may be more permissive to the reparative cues than the adult ones and overall this factor may also contribute to the worst regeneration response in the adult.

We then investigated, as candidates for the pro-regenerative response, the unique M2-conditioned media soluble factors. We explored two candidate cytokines secreted for proof of concept experiments: IL-4, the main M2-expressed cytokine, and IL-6. IL-4 is a cytokine known to drive macrophages to an M2-like phenotype, and in adult mice, the long-acting IL-4 complex enhances cardiac function when administered after coronary artery ligation by reducing infarct size and improving tissue repair^27,28^. Indeed, IL-4 increased cardiomyocyte proliferation (Fig. 5A,B) and stimulated tube formation and progression of the cell cycle as evaluated by G2/M phase (Fig. 5C–F) in endothelial cells. IL-6 is a cytokine that is not only involved in inflammation and infection responses but also in the regulation of metabolic, regenerative and neural processes. In addition, data show the potential of IL-6 to induce endothelial cell proliferation, migration and sprouting^28,29^. Consistent with these data, we observed that IL-6 increased HUVEC cell cycle progression (Fig. 5C,D). These paracrine type effects are in agreement with a growing body of evidence indicating that candidate molecules, often present in extracellular vesicles (e.g. miRNAs and lipids), may become attractive drug targets to increase the efficacy of adult organ repair/regenerative response post-injury to preserve function^30^.

Altogether, we provided evidence that soluble factors from M2 macrophage-conditioned media display pleiotropic pro-regenerative effects on neonatal target cardiac cells and that the candidate cytokines IL-4 and IL-6 recapitulate, at least in part, components of pro-regenerative response. Further characterization of macrophage immune phenotypes and their secreted molecules may give rise to novel therapeutic approaches for post-natal cardiac repair.

## Methods

### Apex resection surgery

Apex resection was performed as previously described by our group^15^ to evaluate overall gene expression differences in 1-day-old (R1) and seven-day-old (R7) rats. Heart samples were collected one day (R1D1/R7D1) and five days post-procedure (R1D5/R7D5). Paired sham-operated rats were submitted to false surgery and collected at the same time points (S1D1/S1D5 and S7D1/S7D5).

### RNA sequencing-Illumina

For total RNA sequencing, only 1/3 of the heart was collected from P1 and P7 and from paired sham-operated rats D1 and D5 after the procedure. After isolation, total RNA from heart samples was purified using an RNeasy Mini Kit (Qiagen) and analysed by an Agilent 2100 Bioanalyzer (Agilent Technologies). Samples must display RIN scores higher than 9.0 and a 260/280 ratio higher than 1.8 to be considered suitable for library preparation through TruSeq stranded total RNA with a “Ribo-zero” gold kit (Illumina). Libraries were individually quantified through qRT-PCR and sequenced using HiSeq2500 (Illumina) with paired-end reads (2 × 100 cycles) according to the manufacturer’s instructions. Reference values considered were a p-value ≤ 0.05; z-score ≤  ± 1.96; and fold-change ≤  ± 1.3. Data acquired were analysed using bioinformatics tools that filtered low-quality reads using Perl script and mapped validated reads using Bowtie2 v.2.1.0 software^31^, which considered the rat genome^32^. Mapped reads were classified by SAMTools v.0.1.18 software^33^, and read counting was performed using HTSeq-counting v.0.5.4p2. Differential expression analysis was performed by DESEq v.1.12.1^34^ using R-Bioconductor software^35^ and a previously described analysis pattern^36^. In addition, Gene Set Enrichment Analysis (GSEA, Broad Institute) was used to determine significant differences in gene expression according to different biological states. Specific pathway analyses were classified and evaluated considering three main parameters: age at the time of the procedure, procedure and time post-procedure. Gene annotations were performed using Biomart software^37^.

### Ingenuity pathway analysis

Analyses were performed in paired groups considering resected rats and their respective sham-operated rats. Through Ingenuity, it was possible to perform core analysis, which resulted in the main biological functions and molecular processes regarding key canonical pathways. Paired comparisons were performed using reference values such as a p-value ≤ 0.05; z-score ≤  ± 1.96; and fold-change ≤  ± 1.3.

### Real-time-polymerase chain reaction (RT-PCR)

RT-PCR was used to assess macrophage polarization and myofibroblast differentiation and secretion. RNA isolation was performed according to the TRIzol RNA isolation protocol, and the precipitate was resuspended in 100 µl of RNA-free water. After total RNA extraction, 5 µg of RNA was reverse transcribed into cDNA using the SuperScript first-strand synthesis system for RT-PCR kit (Invitrogen, Life Technologies) in a T100 Thermal Cycler (Bio-Rad). Then, the sample concentration was assessed by a spectrophotometer, and the integrity was analysed by Bioanalyzer RNA and through 0.8% agarose gel electrophoresis. Macrophage polarization profiles were assessed using the primers listed below (Table 1). GAPDH was used as housekeeping gene and the results are displayed as 2^-ΔΔCt^_,_ mean ± SEM.

**Table 1:** List of primers used for macrophage polarization and myofibroblast differentiation assessment through RT-PCR.

Gene	Sense Primer (5′-3′)	Antisense Primer (5′-3′)
IL-1β	CCTGTGTGATGAAAGACGGC	TATGTCCCGACCATTGCTGT
TNF-α	ATCGGTCCCAACAAGGAGG	GATAAGGTACAGCCCCATCTGC
NOS2	CCTGTGTTCCACCAGGAGAT	CACCAAGACTGTGAACCGGA
Arg1	TCCAAGCCAAAGCCCATAGA	AGCTTTCCTTAATGCTGCGG
TGF-β	TGAGTGGCTGTCTTTTGACG	CAGGAAGGGTCGGTTCATGT
CCL2	CACAGCTGCTGCTTTCACC	GATGGGCTTCAGCACAGACT
α-SMA	TGGAAAAGATCTGGCACCAC	CTCCGTTAGCAAGGTCGGA
Col1	CATGTTCAGCTTTGTGGACCT	TCCACGTCTCACCATTAGGG
Col3	AGCTTTGTGCAATGTGGGAC	ATCACAGAGGACAGATCGGA

M1 macrophages were confirmed by IL-1β, TNF-α and Nos2 expression, and M2 macrophages were assessed for Arginase 1 (Arg1), CCL2 and TGF-β expression. For fibroblast differentiation and secretion, we used α-SMA, Col1 and Col3 expression.

### Histology

Macrophage infiltration was assessed in both P1 (one-day-old, post-natal 1) and P7 (7-day-old, post-natal 7) rats collected at one day (D1) and five days (D5) post-resection. Hearts were collected, embedded in paraffin and stained using the CD68 (ab31630, Abcam) immunohistochemistry protocol. We tested several antibodies described in the literature for characterization and quantification of macrophages (M1 and M2), but we were not successful in the experiments (iNos, 1:50, ab3523, Abcam; and Manose, 1:100, ab64693, Abcam)^38–41^ .

### Cell cultures

#### Neonatal cardiomyocytes and fibroblasts

Neonatal cardiomyocytes and fibroblasts were cultured to evaluate the effects on proliferation and differentiation. Heart samples from 1-day-old Wistar rats were isolated for cardiomyocyte and cardiac fibroblast cultures with a pre‐plating protocol, as previously described^42^. Neonatal hearts were digested with Collagenase Type 2 (Worthington) digestion solution (126 U/ml) and pancreatin (0.2 mg/ml, Sigma–Aldrich) at 37 °C six times with shaking for 15 min. Finally, cells were plated for 45 min (pre-plating), which separated cardiomyocytes (supernatant) and cardiac fibroblasts (adhered cells). Cardiomyocytes were cultured in laminin-coated plates (Life Technologies) at a confluence of 3.5 × 10^4^ cells/cm^2^ in either P12 (for gene expression) well or Greiner CellStar 96-well black polystyrene plates (for protein expression) and treated with either conditioned media or key cytokines for 24 h under normal and hypoxic conditions (1% O_2_). Cardiac fibroblasts were propagated in DMEM-high containing 10% fetal bovine serum (FBS, Life Technologies) and 1% penicillin/streptomycin (Life Technologies). Experiments were performed on passage 1.

Cardiomyocytes and fibroblast were treated with 20 ng/ml IL-4 and 10 ng/ml IL-6. The treatment was performed for 24 h on cardiomyocytes proliferation protocol and 48 h for myofibroblast-induced differentiation protocol^27,43^.

### Differentiation of cardiac fibroblasts to myofibroblasts

Cardiac fibroblasts were treated with 10 ng/ml recombinant human TGF-beta 1 protein (240-B, R&D Systems) for 48 h.

### Isolation of myofibroblasts cells from the infarcted area

Myofibroblasts from the infarcted area of adult rats were cultured to evaluate the effects on reversing the differentiation phenotype when submitted to different conditions. Hearts were collected from adult rat hearts 15 days after descendent artery ligation for differentiation assays.

Ventricles were placed into PBS, and only the injured fibrotic portion was digested.

The ventricles were minced into small pieces to facilitate digestion and were placed into an enzyme solution of PBS containing 240 U/ml Collagenase Type 2 (Worthington), 1 mg/ml Trypsin (Sigma), 1 mg/ml bovine serum albumin (Sigma) and 10% penicillin/streptomycin. The tube was incubated with shaking at 37 °C for 15 min, and the supernatant was collected and transferred to a conical tube containing FBS. This was repeated at least 8 more times until the remaining pieces were too small to separate from the digestion solution. Cells were centrifuged at 400 × *g* for 5 min. The supernatant was discarded, and the pellets were resuspended in DMEM-high supplemented with 20% FBS and 1% penicillin/streptomycin.

Resuspended cells were placed into a cell culture dish and incubated at 37 °C in the presence of 5% CO_2_. Experiments were performed on passage 4.

Cells were cultured at a confluence of 3.5 × 10^4^ cells/cm^2^ and submitted to either conditioned media or key cytokine treatment for 48 h.

### Human umbilical vein endothelial cells (HUVECs)

HUVECs (Lonza C2517A) were cultured according to the manufacturer’s instructions (EGM Bullet Kit-2, Lonza CC-3162) for endothelial cell cycle proliferation and tube formation assays.

### Cell cycle

The cell cycle was analysed using propidium iodide (PI; Sigma–Aldrich). First, the cell cycle was blocked using starvation culture medium for 24 h, and then the cells were treated with IL-4, IL-6 and IL-4/IL-6 concomitantly for 24 h. Finally, the cells were harvested for cell cycle analysis. For this, cells were washed and fixed overnight in cold ethanol (70%). Fixed cells were washed and reconstituted in 250 μL of buffer (0.1% NP40, 0.2 mg/mL RNase, 0.2 mg/mL PI) and incubated for 30 min at 4 °C. Ten thousand events were collected from each sample in an Accuri BD flow cytometer (Becton Dickson), and the data obtained were analysed in the FCS Express 4 programme (De Novo Software).

### Tube formation assay

Geltrex with a low concentration of growth factor (Life Technologies) was added to a 96-well plate for 30 min at 37 °C for polymerization. HUVECs were plated at a density of 10,000 cells per well and incubated with endothelial cell growth basal medium (Lonza) in the absence of supplementation and with IL-4, IL-6 and IL-4/IL-6 concomitantly. The cells were incubated at 37 °C in the presence of 5% CO_2_. Six hours later, network structures were photographed with an inverted microscope using a 4 × objective, and the total length of tube formation was quantified using ImageJ software and the Angiogenesis Analyzer plugin.

### Bone marrow-derived macrophage culture and polarization (BMDMφs)

Bone marrow from adult Wistar rats (300–350 g) was used for macrophage differentiation and polarization assays. BMDMφs were isolated from both hindlimbs and cultured at a confluence of 6.50 × 10^5^ cells/cm^2^ for seven days by GM-CSF in the culture medium every two days (30 ng/ml, Peprotech). Polarization was performed after seven days by 10 ng/ml IFN-γ (Peprotech) for the M1 profile and 20 ng/ml IL-4 (Peprotech) for the M2 profile. After polarization (48 h), cells were harvested for polarization profile assessment using RT-PCR, and the supernatant was collected and frozen for subsequent use.

### Cytokine membrane arrays

Cytokine membrane arrays (rat cytokine array, #ARY008, R&D Systems) were performed to address M2 exclusively expressed cytokines. A pool of eight different polarization protocols was mixed to a final volume of 1 mL/group of M0- (7-day macrophage differentiation, no polarization), M1- and M2-conditioned media. The protocol was performed and analysed according to the manufacturer’s instructions. Cytokines were individually mapped, and both background and positive control values were marked. Final normalized volumes were clustered, and two comparisons were performed: (1) cytokines that were common for both M1- and M2-conditioned media and that were different from control and (2) exclusive M2 cytokines that were different from M1 and control. The membranes were generated and compared as pairs: macrophage control means vs M2 and M1 vs M2. The M1- and M2-conditioned media were obtained from the combination of independent polarization experiments (n = 7).

### High content screening (HCS) analyses

Immunofluorescence assay was performed using High content screening (HCS) as previously described^42^.

For each screening, cardiomyocyte and fibroblast cultures were were plated on 96 well plates in triplicates or quadruplicate. Cells were fixed and stained for proliferation and differentiation (Tropomyosin, 1:300, T-9283, Sigma-Aldrich; α-SMA, 1:100; Abcam, ab5694; Ki67, 1:300; Abcam, ab16667) overnight. Then, cells were washed and conjugated with secondary antibodies (Alexa Fluor 488, 1:500, A11001; Alexa Fluor 555, 1:500, A21428), and cell nuclei were stained with DAPI (1:500, D1306, Invitrogen Life Technologies). Images were acquired using the Image X Micro High Content Platform (Molecular Devices, USA), and five sites per well. Multiwave Cell Scoring Metaexpress Software and Multiwave Cell Scoring were used for data analysis.

### Statistical analyses

One-way ANOVA with Dunnett’s post hoc test was used for macrophage tissue infiltration and HCS analyses, with a p-value ≤ 0.05 for all comparisons. All statistical analyses were performed using GraphPad Prism 5.0 (GraphPad Software Inc.).

### Ethics approval

The use and care of laboratory animals was performed according to institutional guidelines and was approved by the Ethics Committee on Animal Research of the University of São Paulo (CEUA: 294/12).
